# Scotopic Vision Is Selectively Processed in Thick-Type Columns in Human Extrastriate Cortex

**DOI:** 10.1093/cercor/bhaa284

**Published:** 2020-10-19

**Authors:** Roger B H Tootell, Shahin Nasr

**Affiliations:** Athinoula A. Martinos Center for Biomedical Imaging, Massachusetts General Hospital, Boston, MA 02114, USA; Department of Radiology, Harvard Medical School, Boston, MA 02115, USA; Athinoula A. Martinos Center for Biomedical Imaging, Massachusetts General Hospital, Boston, MA 02114, USA; Department of Radiology, Harvard Medical School, Boston, MA 02115, USA

**Keywords:** cortical column, functional connectivity, magnocellular, parvocellular, scotopic vision

## Abstract

In humans, visual stimuli can be perceived across an enormous range of light levels. Evidence suggests that different neural mechanisms process different subdivisions of this range. For instance, in the retina, stimuli presented at very low (scotopic) light levels activate rod photoreceptors, whereas cone photoreceptors are activated relatively more at higher (photopic) light levels. Similarly, different retinal ganglion cells are activated by scotopic versus photopic stimuli. However, in the brain, it remains unknown whether scotopic versus photopic information is: 1) processed in distinct channels, or 2) neurally merged. Using high-resolution functional magnetic resonance imaging at 7 T, we confirmed the first hypothesis. We first localized thick versus thin-type columns within areas V2, V3, and V4, based on photopic selectivity to motion versus color, respectively. Next, we found that scotopic stimuli selectively activated thick- (compared to thin-) type columns in V2 and V3 (in measurements of both overlap and amplitude) and V4 (based on overlap). Finally, we found stronger resting-state functional connections between scotopically dominated area MT with thick- (compared to thin-) type columns in areas V2, V3, and V4. We conclude that scotopic stimuli are processed in partially segregated parallel streams, emphasizing magnocellular influence, from retina through middle stages of visual cortex.

## Introduction

Human visual perception spans an enormous range (roughly 10 log units) of light intensity (Boff et al. 1986; Stockman and Sharpe 2006). To accurately encode visual stimuli across such a daunting range, it is thought that the visual system relies on multiple mechanisms, each sensitive to different parts of that range. Among these mechanisms, optical variations in pupillary diameter compensate for only a small subset (~1.3 log unit) of this range. Remaining mechanisms for coping with variations in light level are thought to be neural (Westheimer 1965). Within the brain, it is thought that neural sensitivity to low light levels is achieved partly by pooling (summing) neural signals across space and time.

### Scotopic and Photopic Vision

Visual stimuli at the lowest visible light levels (e.g., during a moonless night without artificial illumination) are termed “scotopic.” At the other extreme, stimuli at the highest visible light levels (e.g., in bright sunlight) are “photopic.”

Very broadly, scotopic stimuli activate rod-shaped photoreceptors in the human retina. In fact, a given retinal rod can be activated by a single photon, in optimal conditions (Hecht et al. 1941). At much higher (photopic) light levels, rod responses become relatively saturated, and vision relies more on cone-shaped photoreceptors. At intermediate (“mesopic”) light levels, rod and cone systems both contribute to perception, to varying extents.

For millennia, it has been known that scotopic perception differs strikingly from photopic perception (Stabell and Stabell 2009). For instance, scotopic vision is achromatic (gray level), whereas photopic vision includes color perception. Moreover, scotopic vision is spatially less precise (“blurry”) compared to photopic vision. In fact, *in scotopic conditions,* small visual details are better perceived in peripheral parts of the retina, because rods are absent in the most central 0.6^o^ of the retina (Ahnelt et al. 1987; Curcio et al. 1990). In contrast, high acuity information *in scotopic conditions* is perceived best within the cone-dense center of the retina.

### Brain Organization of Scotopic Vision

In contrast to our understanding of scotopic and photopic processing in the retina, very little is known about how scotopic and photopic information processing is organized in the brain itself, particularly in visual cortex. Almost all prior physiological experiments in visual cortex have been conducted at a single photopic level.

The dramatic differences between scotopic versus photopic perception (see previous section) raise the possibility that scotopic versus photopic brain mechanisms also remain segregated well beyond the retina. This idea is supported at initial stages of the visual system: different types of retinal neurons are differentially activated during scotopic versus photopic vision, extending from rods versus cones at the input level (Baylor et al. 1984; Kraft et al. 1993; Schnapf et al. 1990), through two types of ganglion cells at the retinal output level ((Lee et al. 1997; Purpura et al. 1988), but see study by Grunert (1997) and Lee et al. (1997). However, in the brain, very few studies have tested whether 1) scotopic visual processing takes place within different sets of neurons (as in retina), or instead 2) scotopic and photopic cues are merged and processed together in each neuron within a given brain area.

Those competing hypotheses are directly tested here. At the mesoscopic spatial scale, we first tested whether functional magnetic resonance imaging (fMRI) activity during scotopic versus photopic visual processing is segregated in different cortical columns, within one or more cortical area(s). Following confirmation of that hypothesis, we tested if (and how) such photopic/scotopic segregation is related to the well-studied, partially parallel visual “streams” that are so well-established in photopic vision. In prior photopic experiments in nonhuman primates, such subdivisions include the “magnocellular” versus “parvocellular” (and “koniocellular”) layers within the lateral geniculate nucleus (LGN), the column-scale “blob” versus “interblob” streams in V1, and the multiple types of stripe-shaped columns in V2.

Some evidence suggests that scotopic vision is processed preferentially in magnocellular LGN layers, and in magnocellular-dominated cortical sites (Benedek et al. 2003; Hadjikhani and Tootell 2000; Purpura et al. 1988). However, teleologically, such a segregated magnocellular hypothesis would not benefit from shared mechanisms for processing functionally related visual cues, e.g., shape with motion in other contexts (Kriegeskorte et al. 2003; Murray et al. 2003; Treue et al. 1991).

An alternative hypothesis is that scotopic and photopic visual cues are *merged* (rather than segregated) at early levels of brain. A similar convergence of multiple visual cues has been reported at higher levels in photopic vision (Angelaki et al. 2011; Jastorff et al. 2012; Jellema et al. 2004; Morgan et al. 2008; Orban 2011; Vinberg and Grill-Spector 2008). However, such a “merged” model would be limited insofar as scotopic and photopic processing do not include completely overlapping functions or visual field locations.

Our results revealed the mesoscopic organization of scotopic visual processing, which incorporates complementary advantages of both the segregated and merged mechanisms described above. Moreover, this organization appears to emphasize magnocellular channels, throughout early and middle stages of visual cortex.

## Methods

### Participants

Nine human subjects (four females and five males), aged 22–35 years, participated in this study. Among them, six subjects (three females) successfully participated in all tests, including scanning of scotopically driven visual activity (Table 1). The remaining three subjects participated only in control experiments. All subjects had normal or corrected-to-normal visual acuity based on the Snellen test, and radiologically normal brains without history of neuropsychological disorder. All experimental procedures conformed to NIH guidelines, and were approved by Massachusetts General Hospital protocols. Written informed consent was obtained from all subjects prior to all experiments.

**Table 1:** Subject participation in different experiments

		Subjects								
		SBJ 1	SBJ 2	SBJ 3	SBJ 4	SBJ 5	SBJ 6	SBJ 7	SBJ 8	SBJ 9
Experiment	Scotopic experiment	Y	Y	Y	Y	Y	Y	F	N	N
	Photopic experiment	Y	Y	Y	Y	Y	Y	Y	Y	Y
	Functional connectivity	Y	Y	Y	Y	Y	Y	Y	N	N
	Thin columns localizer	Y	Y	Y	Y	Y	Y	Y	Y	Y
	Thick columns localizer	Y	Y	Y	Y	Y	Y	Y	Y	Y
	MT localizer	Y	Y	Y	Y	Y	Y	Y	Y	Y
	Retinotopy napping	Y	Y	Y	Y	Y	Y	Y	Y	Y

Y: Subject participated; N: Subject did not participate; F: Failure due to technical problems.

### General Procedures

Subjects were scanned in a high-field scanner (Siemens 7 T whole-body system, Siemens Healthcare, Erlangen, Germany) for the main experiments. Each subject was scanned in multiple sessions, including one scan session (840 functional volumes) to localize their thin-type (color-selective) columns, and a second scan session (1056 functional volumes) to localize their thick-type (motion-selective) columns. Area MT (also known as V5) (Dubner and Zeki 1971)) was localized based on a moving versus stationary stimulus contrast. In a third scan session (384 functional volumes), we measured each subject’s evoked response to contrast-reversing checkerboards. All these localization stimuli were presented under photopic conditions.

Seven (of the 9) subjects participated in a fourth scan session (1200 functional volumes), which measured responses to achromatic gratings in scotopic conditions, following dark adaptation (Baker et al. 1959; Boynton and Triedman 1953; Nordby et al. 1984) (see below). Data from one subject were excluded, due to problems with dark adaption and projector malfunction. The seven subjects were also scanned in a fifth scan session (1500 functional volumes) with eyes closed, to measure functional connections. Finally, all nine subjects were also scanned in a sixth scan session in a 3 T scanner (Tim Trio, Siemens Healthcare) for structural and retinotopic mapping. Table 1 lists the experiments conducted for each subject. Additional details of the experiment procedures are listed below.

### Experimental Stimuli and Procedure

#### Scotopic Stimuli

A main experimental goal was to selectively reveal brain sites activated by scotopic stimuli, in comparison to spatially uniform gray field (baseline stimuli) at an equivalent scotopic light level. During these scotopic experiments, all nonexperimental sources of light in the scanning room were taped off with black rubberized fabric (BK5, Thorlabs Inc., Newton NJ). Subjects were dark adapted for at least 15 min, and room lights remained off throughout the subsequent fMRI acquisitions (>1 h).

Stimuli were achromatic square wave gratings (0.2 cycles/degree) moving continuously at 4°/s. Previously we found that high-contrast gratings of similar spatial frequency evoked equivalent activity within thin- and thick-type columns under photopic light conditions (Tootell and Nasr 2017). Here, motion direction was reversed every 6 s to reduce possible effect(s) of motion adaptation. Orientation of the stimuli varied between blocks, in 45^o^ steps. Each run included seven blocks (16 s per block) and each block was followed by 16 s of blank presentation (i.e., baseline). Each run began with a spatially uniform field (16 s duration). Total run duration was 240 s. All scotopic gratings and baseline stimuli were presented at a mean luminance of 5.2 × 10^−5^ cd/m^2^. Pupillary diameter could not be measured during scanning, so retinal illuminance (in Trolands) could not be defined here. Each subject participated in 10 runs per session.

Visual stimuli were presented via a projector (Sharp XG-P25X, 1024 × 768 pixel resolution, 60 Hz refresh rate) onto a rear-projection screen, viewed through a mirror mounted on the receive coil array. Stimulus luminance was set to the maximum possible based on the projector limits, which were reduced (~6.5 log units) by interposition of Wratten neutral density (spectrally balanced) filters (Kodak). MATLAB 2018a (MathWorks, Natick, MA, USA) (RRID: SCR_001622) and the Psychophysics Toolbox (Brainard 1997; Pelli 1997) were used to control the stimulus presentation.

#### Photopic Stimuli

Multiple control and localization stimuli were presented at photopic luminance level (mean = 52 cd/m^2^). Again, MATLAB 2018a (MathWorks, Natick, MA, USA) (RRID: SCR_001622) and the Psychophysics Toolbox (Brainard 1997; Pelli 1997) were used to control stimulus presentation.

#### Retinotopic Mapping

The central and peripheral representations of areas V1, V2, V3, and V4 were defined based on retinotopic criteria, using retinotopic mapping procedures described elsewhere (Nasr et al. 2011; Sereno et al. 1995; Tootell et al. 1997; Tootell et al. 1995a). Stimuli were based on either colored images of scenes and face mosaics (8 subjects—scanned in 3 T scanner (see below)) or flashing radial checkerboard (1 subject—scanned in 7 T scanner), presented within retinotopically limited apertures, against a gray background. These retinotopic apertures included wedge-shaped apertures radially centered along the horizontal and vertical meridians (polar angle = 30°), plus a central disk (radius = 0–3°) and a peripheral ring (radius = 5–10°). The isoeccentric representation at 0.6° was based on each subject’s own retinotopic maps of eccentricity (see above) to define the isoeccentricity border at 4°, and extrapolated more centrally by current models of the cortical magnification factor (Sereno et al. 1995).

#### Localization of Thin-Type Columns

As in monkeys, a subset of stimulus-selective activity in human areas V2, V3, and V4 is segregated into two functionally distinct types of columns (thin- or thick-type), each with a stripe-shaped or patchy topography (Dumoulin et al. 2017; Nasr et al. 2016; Nasr and Tootell 2018; Tootell and Nasr 2017). To localize thin-type columns in different blocks, subjects were presented with photopic, isoluminant color-varying, and luminance-varying gratings. All stimuli extended 20 × 20° in the visual field. Grating stimuli were presented at different orientations (either 0°, 45°, 90°, or 135°), drifting in orthogonal directions (reversed every 6 s) at 4°/s. Other details are reported elsewhere (Nasr et al. 2016).

#### Localization of Thick-Type Columns

Thick-type columns were localized by contrasting the activity produced by moving (vs. stationary) gratings. This motion-based localization in human thick-type stripes is consistent with single unit and imaging-based studies in nonhuman primates (NHPs) (DeYoe and Van Essen 1985; Gegenfurtner et al. 1996; Livingstone and Hubel 1987; Tootell and Hamilton 1989).

Motion selectivity was also chosen as a localizer criterion because stimulus motion selectively activates area MT, in both humans (Huk and Heeger 2002; McKeefry et al. 1997; Tootell et al. 1995a; Tootell et al. 1995b; Watson et al. 1993) and NHPs (Dubner and Zeki 1971; Maunsell and Van Essen 1983b). MT was defined as a site in the medial temporal sulcus which responds strongly to the moving versus stationary stimulus contrast. This stimulus also activated additional known motion-selective patches that are located elsewhere, including V3A (centered; near the transverse occipital sulcus) (Tootell et al. 1997), inferior parietal cortex (Pitzalis et al. 2006; Swisher et al. 2007), and a presumptive MSTd-like homolog (Huk and Heeger 2002). Those patches that were outside area MT were excluded from further region-of-interest (ROI)-based analysis.

#### Radial Checkerboards

During one control measurement of activity evoked by photopic stimuli, subjects were presented with a full-screen, achromatic high-contrast-reversing (4 Hz) radial checkboard. In each run, flashing checkerboards were presented four times (each time for 20 s), followed by 45 s of uniform gray presentation. Each run began and ended with 15 seconds of a spatially uniform gray screen (i.e., 290 s per run). Subjects were instructed to maintain fixation on a small centrally presented circle (radius = 0.1°) and to report its color change (red to green or vice versa) by pressing a key on a keypad. Each subject participated in four runs per scan session.

#### Resting-State Functional Connectivity Scans

In these scans, subjects were instructed to keep their eyes closed during the whole scan, but not to sleep. Each scan session consisted of 12 runs, and each run took 256 s. Experimenters talked to the subject between each run to ensure wakefulness.

### Imaging

#### Scotopic Vision Scans

Main imaging experiments were conducted in a 7 T Siemens whole-body scanner equipped with SC72 body gradients (70 mT/m maximum gradient strength and 200 T/m/s maximum slew rate), using a custom-built 32-channel helmet receive coil array and a birdcage volume transmit coil. Voxel dimensions were prescribed at 1.1 mm, isotropic. Single-shot gradient echo-planar imaging (EPI) was used to acquire functional images with the following protocol parameter values: TR = 2000 ms, TE = 26 ms, flip angle = 63°, matrix = 174 × 174, BW = 1512 Hz/pix, echo spacing = 0.79 ms, without phase partial Fourier, field-of-view (FOV) = 192 × 192 mm, 44 oblique-coronal slices, simultaneous multislice [SMS] = 2, acceleration factor *R* = 4 with GeneRalized Autocalibrating Partial Parallel Acquisition (GRAPPA) reconstruction and FLEET-ACS data (Polimeni, et al. 2016) with 10° flip angle.

#### Photopic Vision Scans

In the 7 T scanner described above, functional images were acquired using single-shot gradient-echo EPI with 1.0 mm isotropic voxels with the following protocol parameter values: TR = 3000 ms, TE = 28 ms, flip angle = 78°, BW = 1184 Hz/pix, echo spacing = 1 ms, 7/8 phase partial Fourier, FOV = 192 × 192 mm, 44 oblique-coronal slices, acceleration factor *R* = 4 with GRAPPA reconstruction and FLEET-ACS data (Polimeni et al. 2015) with 10° flip angle.

#### Resting-State Functional Connectivity

The scan sequence used for resting-state functional connectivity was identical to the one used for the main scotopic vision scans (see above), except that each connectivity run included 125 (rather than 120) TRs.

#### Retinotopic Mapping

**Figure 1:**
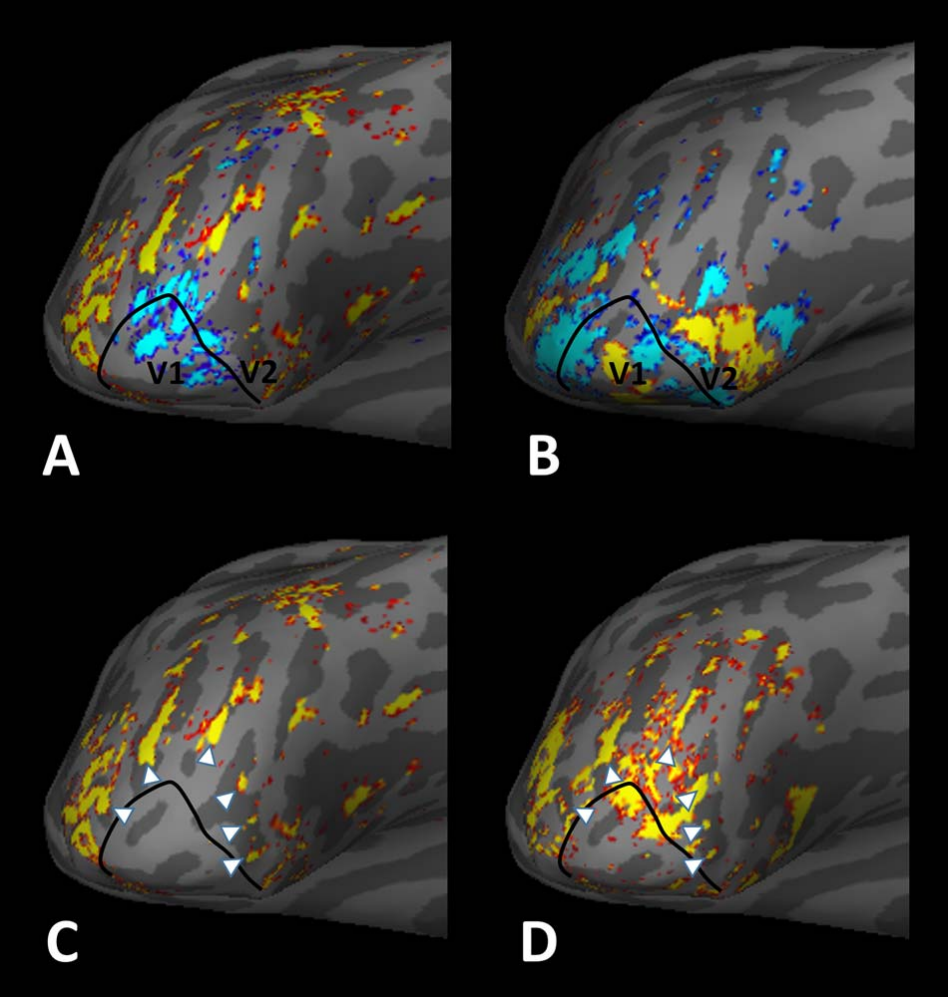
High-resolution fMRI activity evoked by scotopic stimuli, relative to the border of retinotopic visual areas and motion-selective columns in the inflated right hemisphere of one subject, shown from a posterior-ventral-lateral viewpoint. Panel *A* shows BOLD activity produced by a high contrast, moving achromatic grating at scotopic luminance levels, compared to a uniform gray stimulus at equal luminance level. The foveal representation shows negative BOLD responses in V1 and V2. The color activity scale shows *P*-values, with minimum and maximum thresholds = 10^–2.5^ and 10^−5^, respectively. Positive and negative values are indicated by red/yellow and blue/cyan, respectively. Panel *B* shows the activity map evoked by contrasting the response to stimulation of horizontal (red/yellow) versus vertical (blue/cyan) meridians (minimum and maximum thresholds = 0.05 and 10^−3^, respectively). Activity produced by the horizontal versus vertical meridians define the borders between retinotopic visual areas. Panel *C* shows only the positive BOLD changes, to more clearly illustrate the regularly spaced patchy stripes evoked by the scotopic grating (white arrowheads), which occur outside the very central visual field representation in area V2. Panel *D* shows the patchy (sometimes stripe-shaped) activation produced by achromatic moving versus stationary gratings, at photopic levels. In all panels, the black line shows the border between areas V1 and V2, based on independent retinotopic scans (e.g., panel *B*). The location of the white arrowheads (from panel *C*) is duplicated in panel *D*.

For practical reasons, the retinotopic scans were conducted using a 3 T Siemens scanner (Tim Trio) and a Siemens 32-channel receive coil array, for all but one subject. For the remaining subject, whose data were used for illustration purposes (Fig. 1), the retinotopic scans were conducted at 7 T, using the high-resolution procedures described above for photopic vision scans.

At 3 T, the retinotopic data was acquired using single-shot gradient-echo EPI. Voxels were prescribed at 3.0 mm (isotropic) using the following protocol parameters: TR = 2000 ms, TE = 30 ms, flip angle = 90°, matrix = 64 × 64, BW = 2298 Hz/pix, echo spacing = 0.5 ms, no partial Fourier, FOV = 192 × 192 mm, 33 axial slices covering the entire brain, and no acceleration.

#### Structural (Anatomical) Scans

Data were acquired using a 3D T1-weighted magnetization-prepared rapid acquisition with gradient echo sequence with the following parameter values: TR = 2530 ms, TE = 3.39 ms, TI = 1100 ms, flip angle = 7°, BW = 200 Hz/pix, echo spacing = 8.2 ms, voxel size = 1.0 × 1.0 × 1.33 mm^3^, FOV = 256 × 256 × 170 mm^3^.

### Data Analysis

Functional and anatomical magnetic resonance imaging data were preprocessed and analyzed using FreeSurfer and FS-FAST (version 6; http://surfer.nmr.mgh.harvard.edu/) (RRID:SCR_001847) (Fischl 2012). For each subject, inflated and flattened cortical surfaces were reconstructed based on the high-resolution anatomical data (Dale et al. 1999; Fischl et al. 2002; Fischl et al. 1999). All functional images were corrected for motion artifacts. 3 T functional data, used for retinotopic mapping, were spatially smoothed (Gaussian filtered with a 5-mm full width at half-maximum [FWHM]). However, no spatial smoothing was applied to the imaging data acquired at 7 T (i.e., 0 mm FWHM).

For each subject, functional data from each run were rigidly aligned (6 DOF) relative to his/her own structural scan, using rigid Boundary-Based Registration (Greve and Fischl 2009; Yacoub et al. 2008). No distortion correction based on B_0_ field mapping was applied, because compared to other cortical regions (e.g., anterior temporal and/or orbitofrontal), the occipital visual areas (i.e., our main regions of interest) show less spatial distortion (Renvall et al. 2016). Occipital visual areas show smaller vulnerability to pulsatility artifacts, compared to subcortical areas. Previous studies (Enzmann and Pelc 1992; Poncelet et al. 1992) have estimated this effect to produce approximately 90 microns of tissue displacement in the occipital lobe of humans, in vivo, under normal physiological conditions. Given our nominal voxel size (i.e., 1.0 or 1.1 mm iso), this factor should not have significantly affected our measurements.

For functional data, a standard hemodynamic model based on a gamma function was fit to the fMRI signal to estimate the amplitude of the BOLD response. For each individual subject, the average BOLD response maps were calculated for each condition. Then, voxel-wise statistical tests were conducted by calculating stimulus contrasts based on a univariate general linear model. The resultant significance maps were projected onto the subjects’ anatomical volumes and reconstructed cortical surfaces.

### Specific Data Analysis and Tests for 7 T Data

To reduce the impact of pial veins on evoked activity maps (De Martino et al. 2013; Koopmans et al. 2010; Nasr et al. 2016; Polimeni et al. 2010) for all high-resolution functional data collected in the 7 T scanner, activity was sampled from the deepest cortical layer(s). Specifically, for each subject, the gray-white matter (deep) interfaces were defined based on each subjects’ own high-resolution structural scans using FreeSurfer (Dale et al. 1999; Fischl et al. 2002; Fischl et al. 1999).

To measure and quantify the similarity between the scotopically-driven activity within deep versus superficial layers, as expected from a columnar organization in visual cortex, we also generated the gray matter superficial (pial) surface for all subjects using FreeSurfer (Dale et al. 1999; Fischl et al. 2002; Fischl et al. 1999). For one subject, to better show the variation of the activity maps across cortical layers (Fig. 7), an intermediate “mid-gray” surface was also generated at 50% depth of the local gray matter (Dale et al. 1999). Subsequently, the percent fMRI signal change was calculated for those functional voxels that intersected the gray-white matter interface, mid-gray, and superficial layers. The resultant values were projected onto the corresponding vertices of the surface mesh.

The extent of spatial overlap was measured 1) between scotopically-driven activity versus motion-selective activity (used to localize thick-type columns), and 2) between scotopically-driven activity versus color-selective activity (used to localize thin-type columns) by counting those vertices that showed an overlapping and significant response across a wide range of thresholds. Specifically, this test was repeated as the significance threshold was varied for the three activity maps, between *P* = 10^−2^ and *P* = 10^−10^. As a further control, the measured values were normalized by measuring the extent of overlap when the organization of vertices in one map was randomly misaligned (i.e., spatially “shuffled”) and calculating the percentage ratio between the measured overlap in intact versus shuffled conditions. These measurements were conducted independently for V2, V3, and V4 (Fig. 9).

### Resting-State Functional Connectivity Analysis

Details of this analysis are similar to those reported previously (Nasr et al. 2016; Tootell and Nasr 2017). Briefly, after preprocessing (see above), for each subject, we removed sources of variance of noninterest, including all motion parameters (measured during the motion correction procedure), the global signal, the mean signal from the portion of ventricles that were included in the acquired EPI slices, and the mean signal from a region within the deep cerebral white matter. Then, we extracted the mean BOLD signal time course from the area MT, as localized for each subject based on his/her own activity in response to moving versus stationary stimuli (see above). The correlation coefficient was computed for the resultant time course against the preprocessed resting-state time course data, from every voxel from the same hemisphere. The correlation coefficients were then averaged and compared across the regions of interest, including thin- and thick-type columns in the peripheral portions of areas V2, V3, and V4 (see below). Here again, this analysis was restricted to those voxels that intersected the white-gray boundary (i.e., the lower cortical depths).

### Regions of Interest

All ROIs were defined on the cortical surfaces. The borders of ROIs surrounding V1, V2, V3, V3A, and V4 were defined for each subject based on her/his own retinotopic localizers (see above). In V2, V3, and V4, the location of the thin- and thick-type stripes/columns was defined based on the results of independent localizing scans, described above. The few sites showing overlapping selectivity for both color and motion were excluded from all ROIs. Thin and thick stripes were generally difficult to distinguish in the representation of the central visual field, due to competing activity arising from the centrally located fixation target. Accordingly, those central representations were excluded from further analysis in the mesoscopic scale analyses.

### Statistical Analysis

Statistical tests were based on repeated measures analysis of variance (ANOVA). When necessary (based on the Mauchly test), results were corrected for violation of the sphericity assumption using the Greenhouse–Geisser method. We did not observe any between-hemisphere difference (i.e., laterality). Accordingly, in all ROI analyses, data from both hemispheres were averaged together to increase the signal to noise ratio. All statistical analyses were conducted using MATLAB (2018a) (MathWorks, Natick, MA).

**Figure 2:**
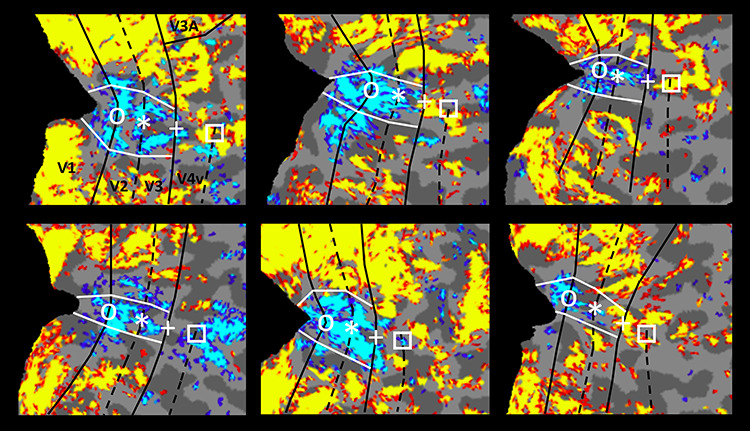
Negative BOLD signals evoked in the foveal representation in all six subjects tested. Each panel shows a flattened patch of the right hemisphere of one subject, otherwise similar to the inflated cortical surface format shown in Figure 1. Data in the top-left panel corresponds to the hemisphere shown in Figure 1*A*. In all panels, the foveal representations are indicated with a black circle at the V1-V2 border, an asterisk at the V2-V3 border, a plus sign at the V3-V4 border, and a square at the anterior border of V4. Retinotopic representations of the vertical meridian borders (e.g., the border between V1/V2 and V3/V4) are indicated with solid black lines, and the horizontal meridians (e.g., the border between V2V3) are represented by a dashed black line. The white lines are estimates of the isoeccentricity representation at 0.6^o^ eccentricity (i.e., the rod-free foveal representation in the retina). For all maps, minimum and maximum thresholds are set at *P* = 0.05 and *P* = 10^−3^, respectively.

To quantify the reliability of activity maps evoked within and across scan sessions during the scotopic light condition, we measured the fMRI signal change evoked by the contrast of interest, for each vertex within the ROI. Subsequently, we tested for a significant correlation between these values either 1) across two scan sessions (for one subject (Fig. 4)) or 2) between the first versus the second half of a scan session (for all subjects (Figs 5 and 6)). We also used the same method to quantify the similarity between vertex-wised activity patterns (evoked by scotopic stimulation) measured within deep versus superficial cortical layers, for all subjects.

It could be argued that the potential correlation between sessions, runs and/or layers might be complicated by nonindependence of activity in adjacent vertices. Thus, we also used a stricter test: for each subject, we randomly selected 10% of vertices, and measured the level of correlation between their activities across the two sessions. This corresponding correlation coefficient was compared relative to the chance level, defined as the level of correlation between the two sessions after randomly misaligning (i.e., spatially “shuffling”) the vertices in one region (i.e., either V2 or V3) relative to the other. We repeated this test 10 000 times for each subject and reported the probability of finding a correlation coefficient that was less than the correlation coefficient of misaligned vertices (i.e., the null hypothesis).

## Results

### Scotopic Activity: Cortical Variations with Eccentricity

Across the human retina, the distribution of rods differs strikingly to that of cones. Rods are entirely absent in the center of the retina—where cones are most densely packed. This central concentration for cones is progressively reversed at greater eccentricities, where rods comprise up to 95% of peripheral photoreceptors (Ahnelt et al. 1987; Curcio et al. 1990). Here we tested whether this scotopically-driven bias in retina is likewise reflected in the central versus peripheral representations in retinotopically organized human visual cortex, using fMRI at high spatial resolution. FMRI activity was evoked by presenting an achromatic, high-contrast square wave grating, compared to a spatially uniform field at equivalent mean luminance (5.2 × 10^−5^ cd/m^2^) (see Methods). For each subject, retinotopy was mapped using independent stimuli.

**Figure 3:**
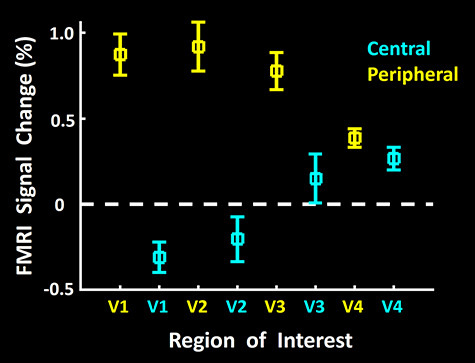
Amplitude of fMRI responses to scotopic gratings in the representations of central versus peripheral visual fields, measured relative to a spatially uniform gray screen (i.e., baseline), in areas V1, V2, V3, and V4. Evoked activity in response to gratings limited to the central (radius = 0–3°) and peripheral (radius = 5–10°) eccentricities is indicated in cyan and yellow, respectively. Consistent with the retinal distribution of rods versus cones, and the maps in Figures 1 and 2, BOLD responses increased significantly in peripheral representations in the most retinotopically organized areas V1 and V2, but BOLD response decreases were found at central representations. Analogous peripheral versus central differences were reduced but remained significantly higher in area V3, but not significantly in V4. Error bars represent one standard error of the mean.

The results confirmed an eccentricity-dependent organization of scotopically driven activity, consistent with the organization of rods in the retina, in all subjects (Figs 1, 2, and 3). As demonstrated in the evoked activity maps of all 6 subjects, in the most retinotopically precise cortical areas (i.e., V1 and V2), strong positive activation was produced by the scotopic stimuli in the stimulated peripheral representation of the visual field (Figs 1 and 2). In contrast, in the central representation, we found that the evoked BOLD signal in response to the scotopic grating was *not* higher than that produced by the baseline activity—in fact, the evoked BOLD signal was negative (i.e., the evoked activity was weaker than the baseline) in areas V1 and V2. This negative activity often extended into area V3 (Fig. 2). This central versus peripheral difference in activation supported our assumption that the “scotopic” stimulus did selectively activate rods (compared to cones) in the retina.

We found similar results based on application of a conventional ROI analysis (see Methods) to the amplitude of fMRI activity evoked by the stimuli presented under scotopic light conditions, in the cortical representation of peripheral (radius = 5–10°) versus central eccentricities (radius = 0–3°), in areas V1, V2, V3, and V4 (Fig. 3). Consistent with the activity maps (Figs 1 and 2), this analysis showed significant evoked activity within the cortical representation of the peripheral (but not the central) visual field, in visual areas V1 and V2 (Fig. 3). This peripheral bias became weaker in area V3, and disappeared in area V4. A two-way repeated measures ANOVA (Visual Field (central vs. peripheral) and Area (V1 vs. V2 vs. V3 vs. V4)) yielded significant effects of Visual Field (F(1, 5) = 20.21, *P* < 0.01) and Visual Field × Area (F(3, 15) = 21.05, *P* < 0.01). These differences between central versus peripheral selectivity across V1, V2, V3, and V4 are consistent with known differences in retinotopic precision due to increased receptive field size and scatter from lower through higher cortical tiers in macaques (Maunsell and Newsome 1987) and analogous pRF measurements in humans (Harvey and Dumoulin 2011; Smith et al. 2001).

**Figure 4:**
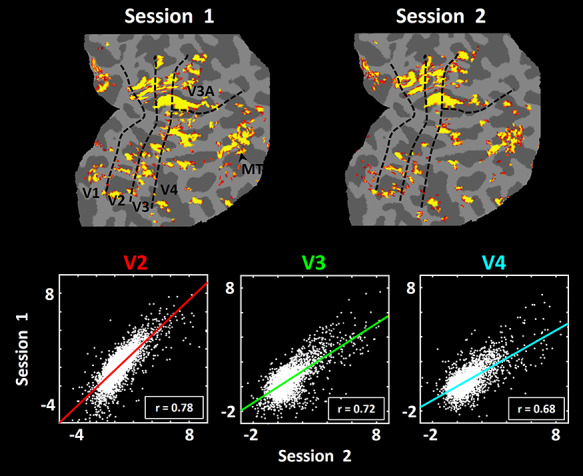
Reproducibility of the evoked activity in response to scotopic stimuli *across* two scan sessions. The upper panels show two activity maps from one subject, both evoked by the scotopic stimuli, acquired 60 days apart. Each session included 1200 functional volumes (see Methods). In both maps, minimum and maximum thresholds are set to *P* = 10^–2.5^ and *P* = 10^−5^, respectively. Borders of the retinotopic visual areas (black dashed lines) were defined based on the subject’s own functional data, acquired in a separate scan session (see Methods). The lower three panels show the correlation in activity across these two sessions, from all surface vertices within area V2 (red; left panel), V3 (green; middle panel) and V4 (cyan; right panel). Each dot represents the activity in one vertex across two different sessions. The correlation *r*-values are furnished in the bottom right corner of each scatter plot.

**Figure 5:**
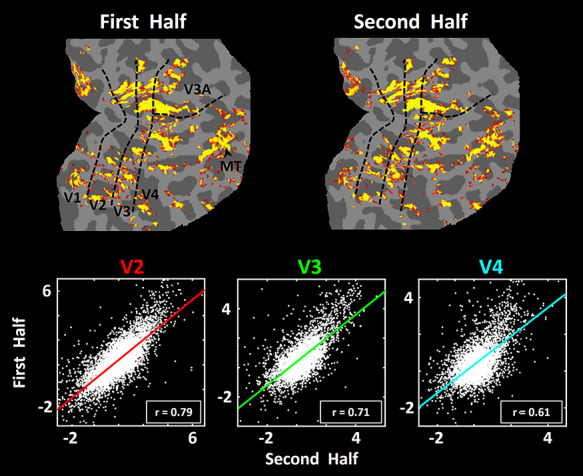
Reliability of the evoked activity in response to scotopic stimuli within one scan session (first vs. second half of the session), in one subject. In both maps (top row), minimum and maximum thresholds are set to *P* = 0.05 and *P* = 10^−3^, respectively. The lower three panels show the correlation in activity across the first versus the second half of the scan session, from all vertices within area V2 (red, left panel), V3 (green, middle panel) and V4 (cyan, right panel). Other aspects of the figure are similar to those in Figure 4.

The scotopic stimulus also produced significant activation in human area MT, in all subjects (mean ± S.D.; 0.72 ± 0.36)(t(5) = 4.87, *P <* 0.01), consistent with our prior study based on conventional fMRI (Hadjikhani and Tootell 2000). The MT activity appeared patchy (Figs 4 and 5) in human MT, presumably reflecting the patchiness in activity shown previously in nonhuman primates to similar stimuli, based on other techniques (Born and Tootell 1992; Geesaman et al. 1997).

In macaques, area MT receives a strong (albeit mostly multiple-stage) input from magnocellular (compared to parvocellular) neurons in LGN in primates (Maunsell et al. 1990; Maunsell and van Essen 1983a; Movshon and Newsome 1996; Nassi and Callaway 2006; Nassi et al. 2006; Sincich et al. 2004). Thus, this scotopic response in human MT is consistent with the hypothesis of a magnocellular-stream bias in scotopic activity, as suggested in macaque LGN (Purpura et al. 1988). The results also showed significant evoked activity in visual area V3A (0.42 ± 0.31) (t(5) = 3.27, *P* = 0.02), which may reflect significant magnocellular influence in that area, as suggested previously (Tootell and Nasr 2017).

### Reproducibility Across Sessions

At scotopic light levels, our hypothesis predicted that stimulus-driven fMRI responses are relatively lower in amplitude, compared to stimulus-driven responses to comparable photopic stimuli (see the photopic tests below). To measure the reproducibility of the evoked activity, we scanned one subject twice (in two different days, 2 months apart) while presenting the same stimuli during the scotopic light condition. As shown in Figure 4, at the levels of signal averaging activity used here (1200 functional volumes per session), maps of scotopically driven activity were robust and reproducible across the two sessions. Consistent with this finding, we found a significant correlation between the vertex-based activity patterns evoked within V2, V3 and V4 during the first versus second scan sessions. This correlation was significantly higher than the correlation between sessions, when the vertex-based activity maps were shuffled randomly (*P <* 10^−3^) (see Methods). Thus, despite the generally lower amplitudes, the scotopically driven activity maps were quite reproducible, like the activity maps evoked by photopic stimuli in prior studies (Nasr et al. 2016; Nasr and Tootell 2018).

### Reliability Within A Session

**Figure 6:**
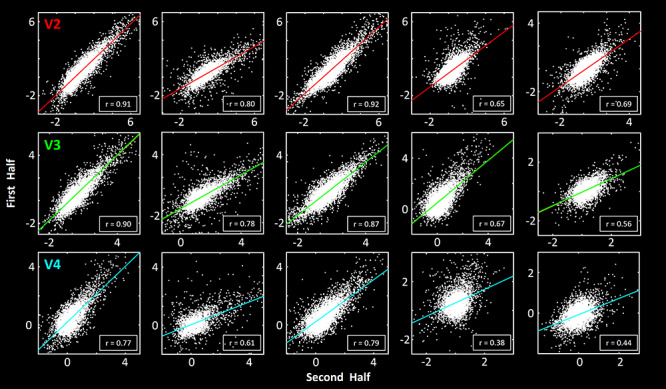
Within-session (first vs. second half) correlation in the five remaining subjects across visual areas V2, V3, and V4. Other figure details are similar to those in the lower three panels in Figure 5.

In these experiments, we acquired scotopically driven activity after 15 to 75 min of dark adaptation, that is, between 85 and 100% of maximum dark-adapted sensitivity. To test for response stability within a session (i.e., reliability), we tested the correlation between the vertex-based scotopically driven activity maps acquired within the first versus the second half of the session. For example, Figure 5 shows the correlation in the evoked activity in one individual subject during “early” (first half of the scan session) versus “late” (second half of the scan session) runs. We consistently found that activity during these two intervals was highly correlated with each other, in all tested visual areas. Here again, this correlation was significantly higher compared to results after the vertex-based activity patterns were shuffled randomly (*P <* 10^−3^). Similar results were also found in sessions in the other five subjects (Fig. 6).

### Radial Organization of Scotopic Activity

**Figure 7:**
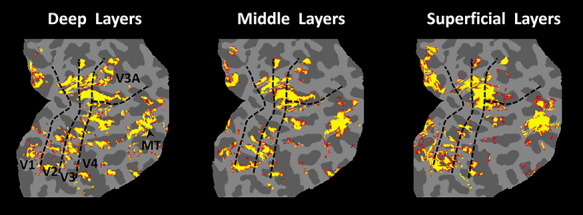
Maps of scotopically driven activity, sampled at different cortical depths, in one subject. In different panels, activity was sampled from voxels that intersected (and were centered on) the white-gray boundary, including layer 6 (left panel), from the middle of the cortical depth, including layer 4 (middle panel), and from the cortical surface, including layer 1 (right panel). Activity in the middle and right panels is color-scaled with minimum and maximum at *P* = 10^−6^ and *P* = 10^−12^, respectively. In the left panel, partly to compensate for the partial volume effects including the white matter, corresponding values are *P* = 10^−2^ and *P* = 10^−4^. Despite this difference in the level of significance, the overall pattern of scotopically driven activity remained similar across the cortical layers, as expected from a columnar organization.

Next, we tested whether scotopic BOLD activity was radially elongated (broadly, “columnar”), consistent with many other columnar arrangements reported within visual cortex in nonhuman primates. Specifically, we sampled and compared the scotopic activity at different cortical depths (layers), ranging from deep (i.e., intersecting the white-gray matter boundary) versus middle versus superficial (intersecting the pia). Despite the expected blurring near the cortical surface arising from pial vasculature (De Martino et al. 2013; Koopmans et al. 2010; Nasr et al. 2016; Polimeni et al. 2010), activity maps across cortical layers remained similar to each (Fig. 7). In all subjects, we also found a significant correlation between vertex-based activity patterns evoked within deep versus superficial layers in V2 (*r* > 0.37), V3 (*r* > 0.24) and V4 (*r* > 0.20), which was significantly higher, compared to the correlation between vertex-based activity patterns when maps were shuffled randomly (*P <* 10^−3^; see Methods). Thus, these results supported the hypothesis that the 3D shape of the scotopically driven BOLD “patches” is elongated along the cortically radial axis. These results supports the hypothesis that the scotopically driven activity is broadly “columnar,” similar to the columnar elongation that was quantified further in the thin- and thick-type columns in V2 and V3 (Nasr et al. 2016).

### Tangential Organization of Scotopic Activity

**Figure 8:**
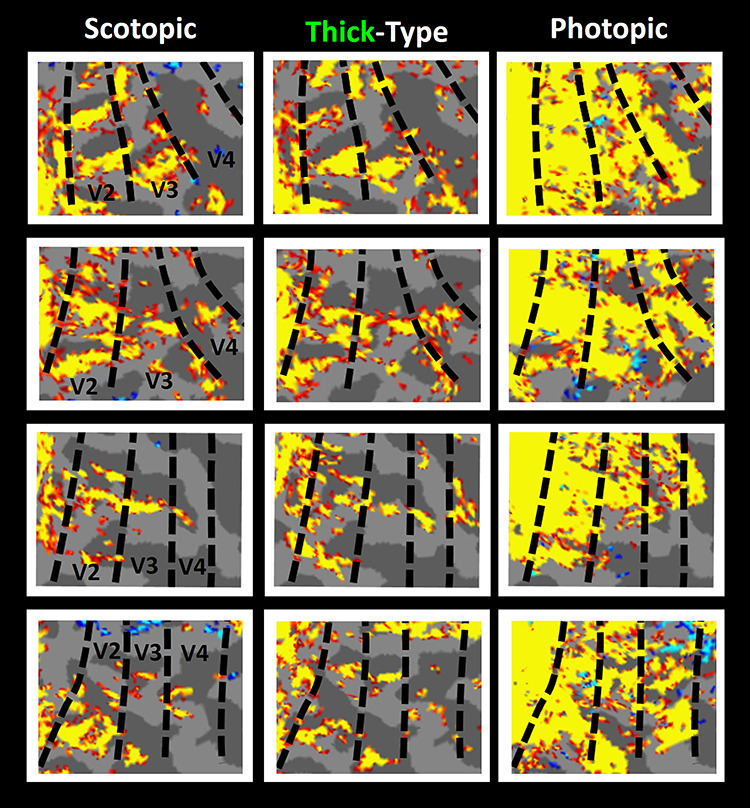
Magnified maps of the fMRI activity in four hemispheres (top through bottom rows), in response to scotopic gratings (left panels), photopic moving versus stationary gratings used to localize thick-type columns (middle panels), and photopic contrast-reversing radial checkerboards (right panels). In all panels, the minimum *P*-value is set higher than *P* < 0.05. Borders of the retinotopic visual areas were defined for each subject based on his/her own functional data, acquired in a separate scan session (see Methods).

At the fine spatial scale sampled here (1.1 mm^3^ iso), the scotopic stimuli selectively activated patchy activity within V2 and V3, when contrasted with activity evoked by the spatially uniform gray stimulus of equivalent (scotopic) mean luminance (e.g., Fig. 1*A*). In V2 and V3, this patchy activity often had stripe-shaped topography. Given the well-established functional columnar subdivisions in monkey V2 (Horton 1984; Livingstone and Hubel 1982; Tootell et al. 1983; Ts'o et al. 2001) and V3 (Tootell et al. 2004) and V4 (Tanigawa et al. 2010), and the perhaps analogous functional subdivisions in human areas V2, V3, and V4 (Dumoulin et al. 2017; Nasr et al. 2016; Nasr and Tootell 2016; Nasr and Tootell 2018; Tootell and Nasr 2017), we wondered whether these *scotopically* driven sites colocalized with either thick- or thin-type columns, which are typically (and here) localized in *photopic* conditions (see Methods). Several different relationships are possible: the scotopic columns could be 1) organized randomly to both thick- and thin-type columns, or alternatively, systematically linked with either 2) thick- or 3) thin-type columns, both localized at photopic light levels. Observations suggested that the scotopically driven columns might preferentially overlap with the thick-type columns (Figs 1 and 8). To quantitatively test this observation, we used two different approaches.

### Scotopic Activity versus Thin- and Thick-Type Columns: Spatial Overlap Analysis

**Figure 9:**
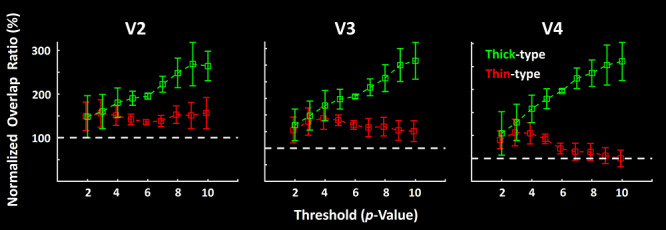
Spatial overlap between the scotopically driven activity and thin- versus thick-type columns. In each panel, the Y-axis shows topographical overlap between the fMRI activity in response to scotopic stimuli (relative to spatially uniform gray display of equal scotopic luminance) and thick- (green) versus thin-type (red) columns, in three extrastriate areas: V2 (left panel), V3 (middle panel), and V4 (right panel). For each subject, the level of spatial overlap is normalized by calculating the percentage ratio between the number of overlapping vertices in intact versus randomly shuffled maps (see Methods). The X-axis shows the level of overlap across different threshold levels. In all three areas, scotopically driven activity preferentially overlapped thick-type (relative to thin-type) columns, at thresholds of *P* < 10^−5^ and increasingly through the highest threshold that could be reliably measured (*P* < 10^−10^). Error bars represent one standard error of mean.

First, we measured the extent of overlap between the activity evoked by scotopic stimulation, relative to that in thick versus thin-type columns across areas V2, V3, and V4 (Fig. 9). Since the level of overlap varies with threshold, this analysis was conducted independently across a wide range of thresholds. Also, at a given threshold level, the level of overlap may vary with the shape and/or surface area occupied by the thick versus thin-type columns. Accordingly, all overlap measurements were normalized relative to the condition in which the activity in vertices assigned to thick- and thin-type columns were spatially shuffled (see Methods). Data from areas MT and V3A were excluded from this analysis because color-selective activity (used to localize thin-type columns) is effectively absent in these areas, in both macaques (Conway et al. 2007; Conway and Tsao 2006; Riecansky et al. 2005; Seidemann et al. 1999; Thiele et al. 2001) and humans (Tootell and Nasr 2017).

In areas V2, V3, and V4, mutually interdigitated thick- and thin-type columns have been demonstrated in humans (Nasr et al. 2016; Nasr and Tootell 2018; Tootell and Nasr 2017). Here, we found greater overlap between scotopically evoked activity in thick-type (compared to thin-type) columns, in all three areas, at commonly used threshold levels (e.g., *P <* 10^−5^), increasing through all higher thresholds at which both thin- and thick-type activity could be measured (through *P*-values of 10^−10^) (Fig. 9). Statistically, application of a three-way repeated measures ANOVA (Column Type (thick- and thin-type), Threshold (2 vs. 3 vs. … vs. 10) and Area (V1 vs. V2 vs. V3 vs. V4) showed significant effects of Column Type (F(1, 5) = 9.30, *P* = 0.03), Area (F(2, 10) = 6.87, *P* = 0.03), and Threshold × Column Type (F(8, 40) = 8.61, *P* = 0.03). Importantly, all *P*-values were corrected for violation of the sphericity assumption (see Methods), which could be caused by a correlation between measured values across different threshold levels.

### Scotopic Activity versus Thin- and Thick-Type Columns: Amplitude Analysis

**Figure 10:**
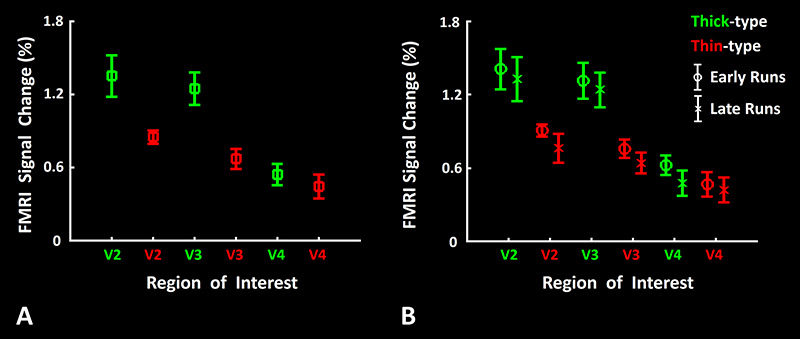
Scotopically driven activity in thin- and thick-type columns. Panel *A* shows the amplitude of fMRI responses to achromatic *scotopic* gratings (relative to uniform gray) in thick- (green) and thin-type (red) columns, in areas V2, V3, and V4. In V2 and V3, responses were significantly higher in thick-type (relative to thin-type) columns. In V4, amplitudes were lower overall, and not statistically different in thick versus thin-type columns. Panel *B* shows a similar comparison, subdivided into early (first half) versus late (second half) runs. No differences were found across early versus late runs. Error bars represent one standard error of mean.

Despite the greater spatial overlap of activity between the scotopically driven sites and thick- (relative to thin-) type columns (Fig. 9), it could be argued that the amplitude of activity might differ in other ways, in comparisons between thin- vs. thick-type columns. To test this, we applied a ROI analysis at the scale of columns. The results showed significantly higher amplitudes within thick- (compared to thin-) type columns in areas V2 and V3 (Fig. 10*A*), consistent with the results in the overlap analysis. In V4, amplitudes decreased overall, and differences between thin- and thick-type columns were not significant. Similar results were also found when we limited our measurements to the ventral portion of area V4 (V4v), in which scotopically driven activity was statistically equivalent in thick- (0.50 ± 0.46) and thin-type (0.34 ± 0.29) columns within V4v. A two-way repeated measures ANOVA (Column Type (thick versus thin-type) and Area (V1 vs. V2 vs. V3 vs. V4)) yielded significant effects of Area (F(2, 10) = 12.00, *P <* 0.01), Column Type (F(1, 5) = 9.16, *P* = 0.03) and Area × Column Type (F(2, 10) = 4.32, *P* = 0.04). Overall, this evidence supported the hypothesis that scotopic stimuli evoke a relatively stronger response in thick- (compared to thin-) type columns.

It could be proposed that activity in thin-type columns might increase with corresponding increases in the duration of dark adaptation. To test that hypothesis, we repeated our ROI analysis after dividing the results from runs in the early (first half) versus late (second half) portion of the scan session. Figure 10*B* shows the amplitude of the evoked activity, measured within thick- and thin-type columns, which remained statistically unchanged between early versus late runs. Consistent with this evidence, a three-way repeated measures ANOVA (Column Type (thick versus thin-type), Area (V1 vs. V2 vs. V3 vs. V4) and Sequence (first vs. second half)) yielded only significant effects of Area (F(2, 10) = 10.81, *P <* 0.01), Column Type (F(1, 5) = 9.57, *P* = 0.03) and Area × Column Type (F(2, 10) = 5.00, *P* = 0.03) without any significant effect of Sequence (F(1, 5) = 0.19, *P* = 0.50), and no interaction between the effect of Sequence and the other independent parameters (*P* > 0.18). This result ruled out the possibility of an increase in the level of thick-type columnar response with increases in the level of dark adaptation.

### Achromatic Photopic Control Test

In V2, thin-type columns can be preferentially activated by specific wavelength combinations, in both humans (Nasr et al. 2016; Nasr and Tootell 2018; Tootell and Nasr 2017) and trichromatic NHPs (Valverde Salzmann et al. 2012; Xiao et al. 2003). In that sense, thin-type columnar activation is broadly “color-selective,” as originally reported (Livingstone and Hubel 1984; Tootell and Hamilton 1989; Tootell et al. 2004; Ts'o et al. 2001; Xiao et al. 2003), but see (Levitt et al. 1994). Because scotopic vision is achromatic, it might be argued that the relative *increase* in scotopic activity in thick- (relative to thin-type) type columns could actually reflect a relative *decrease* of activity in thin-type columns, due to a lack of color sensitivity at scotopic (relative to photopic) light levels.

**Figure 11:**
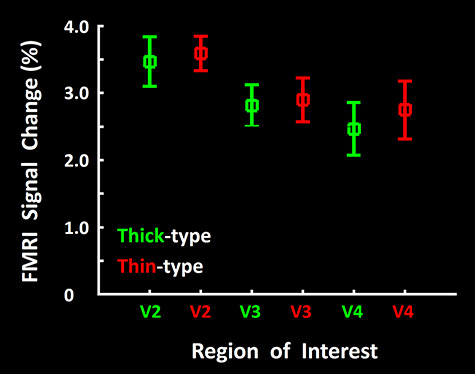
FMRI amplitudes evoked in response to achromatic photopic stimuli (measured relative to uniform gray) in thick- (green) and thin-type (red) columns, in areas V2, V3, and V4. Evoked responses were statistically equivalent between thin- and thick-type columns, in all three areas. Error bars represent one standard error of mean.

We tested this hypothesis by measuring the response to a commonly used stimulus (a stationary, achromatic, contrast-reversing checkerboard pattern) in photopic light conditions, in both thick versus thin-type columns. Although thick- and thin-type columns showed an obvious increase in their level of response to photopic (Fig. 11) compared to scotopic stimuli (Fig. 3), the fMRI amplitude in thick- and thin-type columns remained statistically equivalent. A two-way repeated measures ANOVA (Column Type (thick versus thin-type) and Area (V2 vs. V3 vs. V4)) showed a significant difference between the level of activity evoked across areas (F(2, 16) = 11.38, *P <* 0.01) but no significant difference between the activity evoked within thick- versus thin-type columns (F(1, 8) = 0.17, *P* = 0.69) and/or a significant interaction between Column Type × Area (F(2, 16) = 0.41, *P* = 0.67). These results suggest that the preferential activation of thick-type columns by scotopic stimuli was not secondary to differences in wavelength sensitivity between thick- versus thin-type columns (see Discussion). An analogous response equivalence was found in a different set of subjects (Tootell and Nasr 2017), comparing the activity evoked at photopic levels by achromatic grating stimuli in thick- and thin-type columns in V2, V3, and V4.

### Resting-State Functional Connections between Scotopically Driven Sites

In NHPs, the histologically defined thin- and thick-type columns show segregated “neuroanatomical connections,” within V2, and to/from V2, and with other cortical areas (DeYoe et al. 1994; DeYoe and Van Essen 1985; Levitt et al. 1995; Livingstone and Hubel 1987; Shipp and Zeki 1985). Analogously, previous high-resolution fMRI in humans showed that *functional connections* preferentially interlink alike- (compared with unlike-) type columns (Nasr et al. 2016; Tootell and Nasr 2017) in V2 and V3.

Here we tested a related hypothesis: scotopically driven cortical sites in V2, V3, and V4 (i.e., thick-type columns) are more functionally connected with area MT (another scotopically driven site) and are functionally more connected with each other, compared with sites that do not show such scotopically driven activity (i.e., thin-type columns in areas V2-V4). Neuroanatomical support for this idea includes reports that thick-type columns in macaque V2 are preferentially interconnected with area MT, compared to neuroanatomical connections between thin-type columns to/from MT (DeYoe and Van Essen 1985; Shipp and Zeki 1985).

To test these functional connections in humans, spontaneous fluctuations in high-resolution fMRI signals were extensively measured during the resting state, for each subject (see Methods). Subjects kept their eyes closed during the functional scans. We calculated the resting state functional connectivity between MT (seeded area) and thin- and thick-type columns, in visual areas V2, V3 and V4.

**Figure 12:**
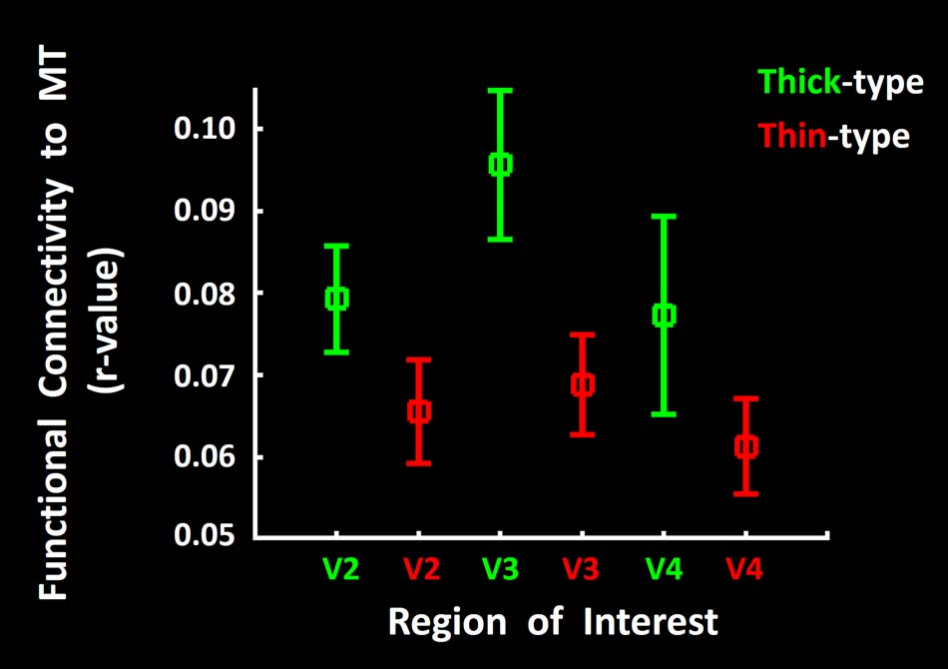
Resting-state functional connections between area MT/V5 and thick versus thin-type columns in areas V2, V3, and V4. In all three areas, fMRI fluctuations in area MT (the seeded area) showed significantly higher correlations with those in thick-type (green) columns, compared to thin-type (red) columns. Error bars represent one standard error of mean.

The results confirmed our hypothesis: BOLD fluctuations in thick-type columns in areas V2, V3, and V4 showed higher correlations (i.e., stronger functional connections) with area MT, compared to those in thin-type columns. A two-way repeated measures ANOVA (Column Type (thick versus thin-type) and Area (V2 vs. V3 vs. V4)) only yielded a significant effect of Column Type (F(1,6) = 7.88, *P* = 0.03) on the level of functional connectivity with area MT (Fig. 12). We also found similar results when we seeded thin- and thick-type columns across visual areas V2, V3, and V4 and used MT as the region of interest (not shown).

## Discussion

Our results indicate that sites of scotopically evoked activity are distributed nonuniformly, in specific columns in early stages of human extrastriate cortex. In V2 and V3, these scotopically activated columns preferentially overlapped thick-type (but not thin-type) columns (Figs 1, 8 and 9). In addition to that preferential spatial *overlap,* response *amplitudes* to scotopic gratings (relative to a spatially uniform baseline) were also higher in thick- compared to thin-type columns, in both V2 and V3 (Fig. 10). Thus overall, both the overlap and amplitude measures suggest a close relationship between scotopic activity in thick- (compared to thin-) type activity, in V2 and V3. In area V4, we found similar (and strong) differences in overlap (Figs 8 and 9), albeit not in amplitude (Fig. 10). This difference between overlap versus amplitude measurements in V4 may be because the simple gratings used for these tests were not optimized stimuli in V4. This interpretation is consistent with previous reports that more complex stimuli are required to produce robust responses in macaque V4 neurons (Arcizet et al. 2008; Desimone and Schein 1987; Gallant et al. 1993; Gallant et al. 1996; Gustavsen and Gallant 2003; Nandy et al. 2013; Pasupathy and Connor 1999, 2002).

These *stimulus-driven* results were complemented by the functional connections measured in the *absence of visual stimuli.* Functional connections were measured while subjects kept their eyes closed, to avoid artifactual correlations of BOLD fluctuations with uncontrolled visual stimulation during the functional scans. We found that BOLD fluctuations in thick-type columns in all three areas of interest (V2, V3, and V4) were preferentially correlated with those in area MT, compared to analogous functional connections between thin-type columns and MT (Fig. 12).

### Segregation of Scotopic Information at the Retinotopic Scale

At the spatial scale of retinotopic gradients and areas, the strong retinal bias of scotopic (rod-dominated) visual processing in more peripheral parts of the human retina (Ahnelt et al. 1987; Curcio et al. 1990) is also reflected in retinotopically organized cortex. Consistent with prior data at lower spatial resolution (Hadjikhani and Tootell 2000), our scotopic stimuli evoked strong positive BOLD responses in peripheral representations of the visual field, without significant positive activity in the central representation of these areas in the most retinotopically organized cortical areas (V1 and V2, and more weakly but significantly, V3). In fact, we found a negative-going BOLD response in this central representation in these areas, in all subjects (Figs 1-3). These negative BOLD responses may reflect hemodynamic (“plumbing”) effects (Goense et al. 2012; Shmuel et al. 2006; Shmuel et al. 2002), or perhaps neural effects such as “filling in” (De Weerd et al. 1995; Mendola et al. 2006). In either event, at high resolution, scotopic stimuli produced positive BOLD responses in the thick-type columns in V2 and V3, within the peripheral (but not the central) representation in retinotopically organized cortex, i.e., consistent with the distribution of scotopically sensitive rods in the retina.

In higher order cortical areas (e.g., V3A and MT), receptive field size in macaques (Maunsell and Newsome 1987) and pRF width in humans (Harvey and Dumoulin 2011; Smith et al. 2001; Tootell et al. 1997) are much larger in visual field representation, and more “scattered” (disordered), compared to those properties in areas V1 and V2 (Hubel and Wiesel 1974). Moreover, V3A and MT are relatively smaller (in cortical surface area), compared to V1 and V2. Both these factors predict that any hypothetical central BOLD decreases would be less evident in V3A and MT, compared to the case in V1 and V2. Effectively, the activity maps in V3A and MT should be more filled in by the decrease in retinotopic order. Such filling in processes could partially account for the negative BOLD signal that we found in central V1 and V2, given the strong feedback connections from higher level to lower level cortical areas (Nassi et al. 2013). The inference of these filling in effects in higher tier areas (e.g., V3, V3A, MT, etc.) (e.g., due to differences in retinotopic precision) is reminiscent of the striking representation of the retinal “blind spot” (optic nerve head) which is strikingly evident in V1 (Awater et al. 2005; Nasr et al. 2020; Tootell et al. 1998), but not reported (likely filled in) in higher visual cortical areas with less precise retinotopic mapping.

### One-Sided versus Mutual Segregation

In macaque monkeys, different types of *photopic* stimuli are known to activate either thin, thick, pale, or multiple columnar “stripes” in V2, in a stimulus-dependent manner (Livingstone and Hubel 1988; Nasr et al. 2016; Peterhans and von der Heydt 1993; Tootell et al. 2004; Tootell and Nasr 2017; Ts'o et al. 2001). Thick- and thin-type columns have been confirmed in human V2 and V3 (Nasr et al. 2016). Similarly, here we found that *scotopic* stimuli preferentially activated the thick-type columns in human V2, and in higher level extrastriate areas. However, we also found that photopically activated sites are distributed relatively uniformly across cortex, including both thin- and thick-type columns (Fig. 11). This uniform distribution of photopically driven activity is consistent with our previous finding that a high-contrast grating (0.27 c/deg) evoked an equivalent response within thin- and thick-type columns across visual areas V2, V3, and V4 (Tootell and Nasr 2017).

Together these results suggest a *one-sided(*partial) segregation of sensitivity to different luminance levels; a columnar organization for scotopic activation but a spatially uniform topographic activation to photopic activation. This one-sided segregation in human cortical imaging is functionally analogous to the one-sided segregation reported from electrophysiological responses recorded in different layers of macaque LGN (Purpura et al. 1988).

As a caveat, it may be noteworthy that the maximum luminance of our photopic stimuli was 105–355 cd/m^2^. Though this experimental light level was within the photopic range (Finkelstein and Hood 1981), it was below the typical photopic levels found outdoors (e.g., 10^4^ cd/m^2^, as found under a clear sky at noon). Thus, it is conceivable that brighter visual stimuli would have preferentially activated thin-type (compared to thick-type) columns—rather than the statistically equivalent activation in thick- versus thin-type columns that we found here. If confirmed, that hypothetical result could support a more classic *mutual* (doubly dissociated) segregation of scotopic versus photopic information.

### Long-Distance Scotopic/Photopic Streams?

It is tempting to infer that the clustering of scotopically driven activity in a given cortical site (e.g., V2, V3, etc.) is interconnected with scotopic clustering in other sites (e.g., ganglion cell, LGN), in partially parallel neural channels extending from retina through LGN through lower and middle levels of extrastriate cortex. However, further research is required to confirm this hypothesis. One challenge is that the neuroanatomical connections between different layers in LGN with hypothetically linked columns in V2 are unresolved, at least through macaque V1 (Sincich and Horton 2002, 2005; Sincich et al. 2010).

### Luminance Contrast versus Light Level

In NHPs, one well-studied functional property of neurons in magnocellular versus parvocellular layers in LGN is a difference in response to variations in contrast sensitivity (contrast = (L_max_—L_min_)/(L_max_ + L_min_), where L = mean luminance). In comparison to parvocellular neurons, magnocellular neurons in LGN are generally more sensitive to low stimulus contrasts (Derrington and Lennie 1984; Kaplan et al. 1987; Kaplan and Shapley 1986). This relatively higher contrast sensitivity in magnocellular layers of LGN has been used to infer a magnocellular-stream influence in additional stages of the visual system, from retinal ganglion cells (Purpura et al. 1988), through specific cortical sites (Blasdel and Fitzpatrick 1984; Sclar et al. 1990; Tootell et al. 1995b; Tootell and Nasr 2017).

Importantly, the physical dimension of light *level* is independent of light *contrast*. Thus, the current demonstration of clustered thick-type columnar activity at low light level does not necessarily conflict with (nor support) earlier reports of higher thick-type activity at low light contrast (Tootell and Nasr 2017). However in vivo, sensitivity to *light level* (e.g., scotopic through photopic) could conceivably interact with the sensitivity to contrast, depending on the neural mechanism(s) by which these two physical dimensions are encoded. In magnocellular LGN neurons, it has even been speculated that the apparent high sensitivity to contrast is secondary to a primary higher sensitivity to light level (Kaplan et al. 1988; Shapley 1990). This question remains unresolved, partly because almost all physiological measurements have been acquired at a single photopic light level.

### Is the Magnocellular Stream Specialized for Scotopic Information?

In NHPs, Purpura et al. (1988) reported that electrophysiological responses in magnocellular LGN were activated by both scotopic and photopic stimuli, whereas responses in parvocellular LGN were activated only by photopic stimuli. The authors suggested that scotopic vision may be preferentially processed in magnocellular- (rather than parvocellular-) dominated cortical sites at higher stages. Consistent with this hypothesis, another study combining psychophysics and electrophysiology concluded that “under scotopic conditions, human visuospatial processing is characteristically predominated by the functional activity of the magnocellular pathways” (Benedek et al. 2003). Consistent with this, a comparative study (Hassler 1966) reported that magnocellular LGN layers are relatively larger in species that are active in scotopic compared to photopic conditions, that is, in nocturnal compared to diurnal ecological niches.

One obvious test site for this scotopic-magnocellular hypothesis is area MT. Much evidence from electrophysiology (Maunsell et al. 1990; Movshon and Newsome 1996) and neuroanatomy (Maunsell and van Essen 1983a; Nassi and Callaway 2006; Nassi et al. 2006; Sincich et al. 2004) suggests that macaque MT is dominated by magnocellular (compared to parvocellular) influences. In humans, a conventional-scale fMRI study (Hadjikhani and Tootell 2000) also suggests that scotopic stimuli activate the human homolog of area MT. In contrast, other ventrally located extrastriate areas (in/near V4v) responded to photopic (but not scotopic) stimuli. In NHPs, direct measurements indicate that neighboring area V4 is relatively more influenced by parvocellular information, compared to MT (Ferrera et al. 1994). Thus, the strong and selective response to scotopic stimuli that we found in human MT (Hadjikhani and Tootell 2000) supports the hypothesis that scotopic stimuli selectively activate a magnocellular “stream.”

At the scale of cortical columns and single neurons, neuroanatomy in NHPs suggests that thick-type columns in V2 are preferentially influenced by magnocellular influences (DeYoe and Van Essen 1985; Shipp and Zeki 1985), which is consistent with analogous inferences in human imaging (Tootell and Nasr 2017). However, other results suggest that macaque thick-type columns may be influenced (sometimes multisynaptically) by both magnocellular and parvocellular LGN cells (Sincich and Horton 2002, 2005; Sincich et al. 2010). In humans, our measurements also support the magnocellular-scotopic hypothesis, in that thick-type (compared with thin-type) columns in V2, V3, and V4: 1) showed preferential functional connections with area MT (Fig. 12), and 2) respond robustly to scotopic stimuli (Figs 4, 5, and 7) (see also (Hadjikhani and Tootell 2000)).

Very generally, it has been proposed that parvocellular processing emphasizes neural *subtraction*, e.g., in service of color opponency and higher spatial resolution. In contrast, magnocellular neurons emphasize neural *summation*, e.g., to maximize sensitivity (Shapley 1990). This generality is consistent with the present data, in that scotopic processing preferentially involves the magnocellular stream, and extensive neural pooling is thought to underlie scotopic visual processing.

### Color Vision and Scotopic Sensitivity

In imaging studies, thin-type columns in V2 are “color-selective,” and respond selectively to variations in wavelength (especially end-spectral) in both macaques (Livingstone and Hubel 1984; Tootell et al. 2004; Ts'o et al. 2001; Valverde Salzmann et al. 2012; Xiao et al. 2003) and humans (Nasr et al. 2016; Nasr and Tootell 2018). This topographical interdigitation of color-selective thin-type columns with scotopic-sensitive thick-type columns is consistent with the current results, because color perception is present only in photopic conditions, not in fully scotopic conditions.

Thus here, an alternative interpretation of the empirical thick-type bias produced by scotopic stimuli in V2 and V3 instead reflects: 1) moderate responses in thick-type columns to the scotopic contours, coupled with 2) *relatively lower* activity in the color selective thin-type columns. According to that interpretation, the apparent activity bias in thick-type columns would be a secondary effect, due to the lack of color in our stimuli.

However, this alternative hypothesis is belied by the data. In response to a typical black-and-white checkerboard stimulus at photopic levels, and in a similar photopic comparison using achromatic gratings (Tootell and Nasr 2017), we found that thin-type and thick-type columns responded essentially equally to each other (Fig. 11). Thus, the clustering of the scotopic responses appears to be a primary effect, rather than a secondary effect of color sensitivity in photopic conditions.

### Motion Sensitivity across Luminance Level

In the human V2/V3, thick-type columns show high sensitivity to both: 1) contrast-reversing, stationary scotopic stimuli (Tootell and Nasr 2017), and 2) moving (vs. stationary) stimuli (see Methods). This sensitivity profile suggests that scotopic sensitivity may be especially linked to motion perception. In support of this idea, one psychophysical study concluded that “movement detection is a very robust process that tolerates extremely low retinal luminance levels” (van de Grind et al. 2000). A different study described a “fast” scotopic system that can resolve temporal frequencies as high as 32 Hz in scotopic conditions, which was attributed to activity in motion-selective area MT (Conner and MacLeod 1977). In another study, perceptual deficits were smaller for motion cues, compared with form cues, as light level was reduced (Burton et al. 2016). In photopic conditions, motion sensitivity is typically highest in the central visual field, whereas in scotopic conditions, sensitivity is highest in the peripheral visual field (i.e., broadly consistent with the distribution of rods in the retina). However, in early studies, both temporal and spatial thresholds decreased relatively monotonically with light level (Barlow 1958; van Nes et al. 1967), without any obvious scotopic-photopic break in the slope of the functions*.* Overall, some (but not all) psychophysical studies suggest a preservation of motion sensitivity at scotopic light levels.

### Potential Limitations

Our results suggest that selective functional connections exist between thick- (compared to thin-) type columns (in V2, V3 and V4) to/from area MT. However, the noninvasive tools that are available for measuring “point-to-point” neuroanatomical connections remain technically limited in humans. To clarify such connections, more direct (and high resolution) measures of human neuroanatomical connections between adjacent visual areas are likely necessary (e.g., see (Attar et al. 2020)).

For the main scotopic experiments, we presented 1-D varying gratings at ~98% contrast. Grating stimuli were chosen to confine the 1-D spatial frequency to a single dominant value, with predictable smaller harmonics, for each orientation presented. The spatial frequency of the gratings (0.2 c/deg) was chosen mainly because both thick- and thin-type columns produced essentially equally strong responses to this spatial frequency, when considered across the three areas targeted, outside the central visual field, in our prior experiments using 7 T, high-resolution BOLD techniques (Tootell and Nasr 2017). In the choice of spatial frequency, we also considered the spatial frequency tuning of area MT in nonhuman primates (O'Keefe and Movshon 1998; Priebe et al. 2003; Priebe and Lisberger 2004; Priebe et al. 2006), and psychophysical findings in humans.

For the photopic experiment, we had previously reported that similar gratings (when presented photopically) evoke a statistically equivalent response within thin- and thick-type columns across visual areas V2-V4 (Tootell and Nasr 2017). Thus, here we measured activity in response to a different stimulus (achromatic checkerboard stimuli) at photopic light levels. Checkerboard stimuli have a spatial frequency spectrum that is even broader than that of a grating (De Valois et al. 1979). In prior studies, such checkerboard stimuli have strongly activated early visual cortical areas, based on fMRI and evoked responses in humans, human psychophysics, and single unit studies in macaque V1 (De Valois et al. 1979).

### Implications and Future Research

Functional differences of neurons in the two most prominent layers of the LGN (and related ganglion cells inputs) are typically listed in terms of empirical, lower level properties, without any unifying teleology. For instance, relative to parvocellular LGN neurons, magnocellular LGN neurons are reported to be more sensitive to variations in luminance contrast (Derrington and Lennie 1984; Kaplan and Shapley 1982, 1986; Kaplan et al. 1988; Purpura et al. 1988), less sensitive to color (Derrington et al. 1984; Kaplan et al. 1988; Lennie and Movshon 2005; Shapley 1990), more sensitive to high temporal frequencies (Derrington and Lennie 1984) and lower spatial frequencies (Derrington and Lennie 1984; Shapley et al. 1981), and to have larger receptive fields at a given eccentricity (Blasdel and Fitzpatrick 1984; Derrington and Lennie 1984; Perry and Cowey 1981). In other words, consistent with its early (precortical) stage within the visual processing hierarchy, the LGN (or its ganglion cell inputs) is not proposed to be especially sensitive to any particular higher order property, e.g., faces, or places, or bodies, or language.

However, the data here suggest that a different organizational principle may exist in LGN, which could potentially unify the known list of otherwise-diverse functional properties. Simplistically: magnocellular neurons may be optimized for scotopic processing, and photopic neurons for photopic processing. For instance, sensitivity to color variations is prominent in photopic perception (and parvocellular neurons)—but absent in scotopic perception (and magnocellular neurons). Further research is required to test this hypothesis.

## Conclusion

Using high-resolution neuroimaging at 7 T, these results provide direct evidence for a mesoscopic (column-scale) dissociation between the processing of scotopic versus photopic vision in human visual cortex. Moreover, the visual activation during scotopic light conditions is consistent with the hypothesis that scotopic vision is preferentially processed in a magnocellular-dominated “stream.” In contrast, activation during mid-range photopic light levels supports the hypothesis that photopic vision relies on both magnocellular and parvocellular processing. More direct tests using higher spatial resolution neuroimaging techniques (e.g., see (Berman et al. 2020)) are required.

## Funding

The National Institute of Health National Eye Institute (grants R01EY026881 and R01EY030434); the Massachusetts General Hospital [the host institution]/Health, Science and Technology [the program within MGH, which is also affiliated with the Massachusetts Institute of Technology (MIT)] Athinoula A. Martinos Center for Biomedical Imaging; NIH Shared Instrumentation (Grants S10RR019371 and S10OD023637 for crucial resources); and Biomedical Technology Research (resource P41EB015896).
